# Cytochrome P450 Monooxygenase/Cytochrome P450 Reductase Bi-Enzymatic System Isolated From *Ilex asprella* for Regio-Specific Oxidation of Pentacyclic Triterpenoids

**DOI:** 10.3389/fpls.2022.831401

**Published:** 2022-03-24

**Authors:** Le Li, Shumin Lin, Yuanyuan Chen, Yaqin Wang, Luhua Xiao, Xiaofang Ye, Lei Sun, Ruoting Zhan, Hui Xu

**Affiliations:** Key Laboratory of Chinese Medicinal Resource From Lingnan (Ministry of Education), School of Pharmaceutical Sciences, Guangzhou University of Chinese Medicine, Guangzhou, China

**Keywords:** *Ilex asprella*, triterpenoid, biosynthesis, CYP, CPR

## Abstract

*Ilex asprella* is a plant from Aquifoliaceae. Its root is commonly used as folk medicinal materials in southern China. The chemical compositions of *I. asprella* are rich in pentacyclic triterpenoids, which show various biological activities and demonstrate a good prospect for drug development. The elucidation of biosynthesis mechanism of triterpenoids in *I. asprella* could lay important foundations for the production of these precious plant secondary metabolites by metabolic engineering. Our previous studies have revealed IaAO1 (a CYP716A210 homolog) responsible for the C-28 oxidation of α- and β-amyrin. Herein, we reported the identification of three more cytochrome P450 monooxygenase genes *IaAO2* (a CYP716A212 homolog), *IaAO4* (CYP714E88), *IaAO5* (CYP93A220), and a cytochrome P450 reductase gene *IaCPR* by using *Saccharomyces cerevisiae* eukaryotic expression system and gas chromatography-mass spectrometry. Among them, the protein encoded by *IaAO2* can catalyze the C-28 oxidation of α-amyrin and β-amyrin, *IaAO4* can catalyze the C-23 oxidation of ursolic acid and oleanolic acid, while *IaAO5* is responsible for the C-24 oxidation of β-amyrin. By introducing three genes *IaAO1*, *IaAO4* and *IaCPR* into *S. cerevisiae.* We constructed an engineered yeast strain that can produce C-23 hydroxyl ursane-type triterpenoid derivatives. This study contributes to a thorough understanding of triterpenoid biosynthesis of medicinal plants and provides important tools for further metabolic engineering.

## Introduction

As a group of important plant secondary metabolites, triterpenoids exist widely in fungi, ferns, monocotyledons, dicotyledons and animals, especially dicotyledons (Liby et al., 2007). These compounds have a wide range of pharmacological activities such as anti-tumor, anti-inflammatory, anti-virus and immune regulation (Nazaruk and Borzym-Kluczyk, 2015; Bao et al., 2018; Lou et al., 2021). However, the structures of triterpenoids are complex and the content of triterpenoids in plants is relatively low. Most of these compounds are mixed with compounds with similar structures. So it is difficult to obtain a large quantity of triterpenoid monomers by plant extraction, separation or chemical synthesis (Moses et al., 2015). Nowadays, with increased understanding of the biosynthetic pathway of triterpenoids, synthetic biology has turned into a promising platform for high production of these high-value natural products.

There are many key enzymes involved in the biosynthesis of triterpenoids. Cytochrome P450 monooxygenases (CYPs), as an important enzyme in the metabolism of plant triterpenoids, directly affect the structural diversity and biological activity of triterpenoids as they participate in the oxidative modification downstream of their biosynthetic pathway. Recently, quite a few CYP genes involved in the oxidation of triterpene skeletons have been identified. For example, CYP88D6 encodes a β-amyrin 11-oxidase catalyzing the sequential two-step oxidation of β-amyrin at C-11 to produce 11-oxo-β-amyrin in the glycyrrhizin pathway (Seki et al., 2008). CYP716A52v2 as a β-amyrin 28-oxidase played a key role in the formation of oleanane-type triterpene biosynthesis in the ginsenoside pathway (Han et al., 2013). CYP716A141 as a β-amyrin 16-oxidase was able to catalyze C-16β hydroxylation of β-amyrin in the platycodon grandiflorus saponin pathway (Tamura et al., 2017). CYP72A68v2 and CYP93E2 were involved in the hemolytic and non-hemolytic sapogenin biosynthetic pathways in *M. truncatula*, mainly the oxidative modification at position 23 and 24 (Fukushima et al., 2013). Despite the identification of more and more CYPs, their oxidative modification mechanism of many sites has not been totally revealed (Seki et al., 2015). There are still great challenges in the functional characterization of CYPs due to the low yield of products, low stability of enzymes and rapid consumption of cofactors (Ohnishi et al., 2006; Rasool and Mohamed, 2016; Liu et al., 2020).

Cytochrome P450 reductase (CPRs) is an important component of cytochrome P450 system (Guengerich et al., 2009). When CYPs works, CPRs is included as the electron donor and the electron transfer reaction between them is also believed to be the rate-limiting step of CYP catalytic oxidation (Rana et al., 2013). Matching CYP with appropriate CPR can improve the catalytic efficiency of P450 system. Recently, glycyrrhetinic acid and 11-oxo-β-amyrin in engineered yeast were greatly improved by co-expressing new CYPs and CPRs from plant sources. This provided a new way for the optimization and regulation of exogenous pathway in yeast (Zhu et al., 2018).

**FIGURE 1:**
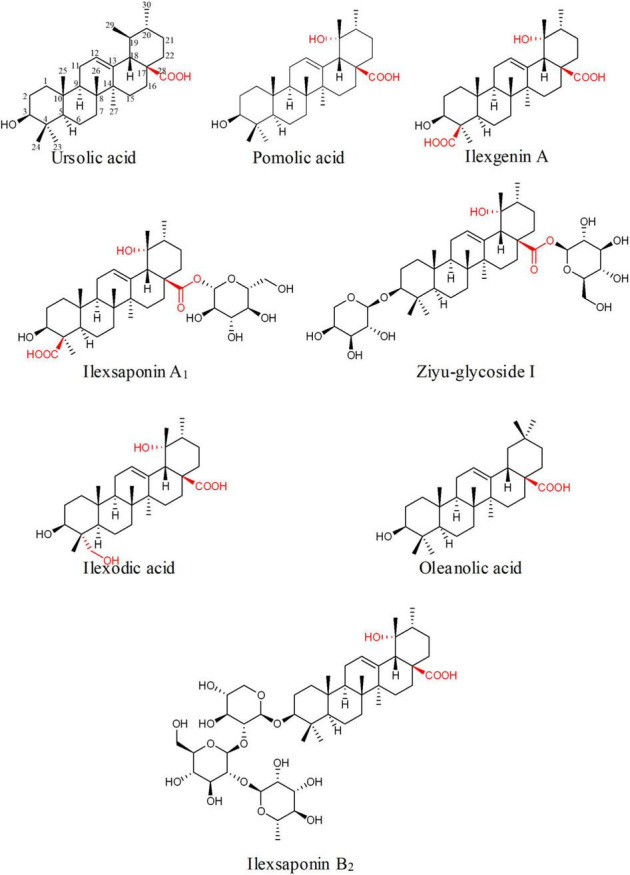
Some simultaneous triterpenoids in *Ilex asprella*. These compounds were described in order to predict the structural characteristics of related compounds and expected related key enzymes. Substituents and substitution positions were indicated in red.

*Ilex asprella* from Aquifoliaceae is popular traditional medicinal materials widely used in Southern China (Li et al., 2012; Yang et al., 2018). The main active components of *I. asprella* are a great variety of ursane- and oleanane-type triterpenoids with oxidation and glycosylation modification (Du et al., 2018; Huang et al., 2019). The oxidative modification occurs at various positions, mainly at C-19, 23, 24, and 28 (Figure 1). These triterpenoids show various biological activities and high clinical application values. Among them, asprellanoside A had obvious antiviral effect determined by *in vitro* anti-HSV-1 activity test (Zhou et al., 2012). Ilexoside O had obvious anticoagulant effect and could decrease the amount of the abdominal aortic blood clots (Taketa et al., 2004). Therefore, it is worthwhile to study the biosynthesis of pentacyclic triterpenoids of *I. asprella*.

Previously, we have obtained the transcriptome of *I. asprella* using RNA-sequencing (GenBank accession number SRP035767) (Zheng et al., 2014). Transcriptome analysis showed that several Unigenes might be related to pentacyclic triterpene biosynthesis. Among them, IaAS1 and IaAS2 were identified as oxidosqualene cyclase, catalyzing the conversion of 2, 3-oxidosqualent to α- and β-amyrin at different ratios. IaAS1 mainly produces α-amyrin, accounting to ≥80% of total amyrin production. IaAS2 mainly synthesizes β-amyrin with a yield of 95% (Zheng et al., 2015). And IaAO1 (a CYP716A210 homolog) catalyzes the C-28 carboxylation of amyrin (Ji et al., 2020). However, genes pertaining to the oxidation at other positions are still unknown. In this study, we reported the identification of three *CYP* genes, involved in oxidative modifications at C-28, C-23 and C-24 positions, and one *CPR* gene *IaCPR*, the cofactor of bi-enzymatic system.

## Materials and Methods

### Materials

α-amyrin and β-amyrin of 98.5% purity were purchased from Sigma-Aldrich (Shanghai, China). Ursolic acid and oleanolic acid of 98% purity were purchased from Energy Chemical (Shanghai, China). Hederagenin of 98% purity was bought from TCI (Shanghai, China). Gypsogenic acid of 95% purity was purchased from TRC (Beijing, China). α-Boswellic acid of 98% purity was purchased from PUSH BIO-TECHNOLOGY (Sichuan, China).

### Sequence Analysis

Unigene or Contig annotated as “cytochrome P450 monooxygenase” was retrieved from transcriptome of *I. asprella*. Only those with a length of more than 1,200 bp, FPKM higher than 50 and *E*-value close to 0 were selected and subjected to phylogenetic analysis together with complete amino acid sequences of already known CYPs randomly selected from NCBI.^1^ As for CPR, Unigene or Contig with full open reading frame annotated as “cytochrome P450 reductase” were retrieved and used to build the phylogenetic tree with known CPRs. The phylogenetic tree was constructed by maximum likelihood method and molecular evolutionary genetic analysis program (MEGA 7.0) (Kumar et al., 2016). And the reliability of evolutionary branches was tested by bootstrap and repeated 1,000 times (Felsenstein, 1985). The properties of deduced amino acid sequences were estimated by using TMHMM Server v2.0^2^ programs. ClustalW and Jalview version 2 was used for multiple sequence alignment (Waterhouse et al., 2009).

### Amplification of Gene Candidates

Total RNA was extracted from the fresh picked young leaves of 5-year-old *I. asprella* using a HiPure Plant RNA Mini Kit (Magen, Guangzhou, China). Using them as templates, cDNA was obtained by TransScript II All-in-One First-Strand cDNA Synthesis SuperMix (TransGen Biotech, Beijing, China), and the gene coding regions were amplified by using the PrimerSTAR high-fidelity DNA polymerase (Takara, Dalian, China). The PCR products obtained were purified and ligated into the vector *pEASY*-T5 and subsequently recombinant plasmids were used to transform *Escherichia coli Trans*1-T1 competent cells using a *pEASY*-T5 Zero Cloning Kit (TransGen Biotech, Beijing, China). The recombinant plasmids were verified by sequencing. All primers used in this study are shown in Supplementary Table 1. Details of strains and plasmids are listed in Supplementary Table 2.

### Construction of Plasmids for Heterologous Expression

In-Fusion primers were designed based on the sequences of *IaAO2* and the yeast expression vector pESC-TRP. After amplification, the coding region of *IaAO2* was ligated into the vector pESC-TRP between *Bam*HI and *Xho*I restriction sites using the In-Fusion HD Cloning Kit (Takara, Dalian, China). The obtained plasmid was named as pT*IaAO2*. Using the same method as described above, the coding region of *IaAO4*, *IaAO5*, and *IaCPR* were ligated into the vector pESC-URA, pESC-TRP and pET32a to give the plasmids named as pU*IaAO4*, pT*IaAO5*, and pET32a*-IaCPR*, respectively. Furthermore, *IaCPR* was inserted between *Eco*RI and *Spe*I restriction sites of the obtained plasmid pT*IaAO2* using In-Fusion cloning technique, resulting in plasmid pT*IaAO2-IaCPR* containing both *IaAO2* and *IaCPR* genes.

### Prokaryotic Expression of Cytochrome P450 Reductase Candidates and Enzyme Assays

The recombinant expression plasmid was transformed into *E. coli* Transetta (DE3), the recombinant protein was induced by 1 mM IPTG and purified by Ni^2+^-NTA chromatography (Qiagen, Germany). SDS-PAGE was performed to assess the protein expression levels and purity. The electron transport activity was measured by the change of optical absorption at 550 nm of reduced cytochrome c and the change of optical absorbance of K3Fe(CN)6 at 424 nm. Absorbance changes were recorded by Microplate Reader (Bio-Rad Laboratories, United States).

### Eukaryotic Expression of Cytochrome P450 Monooxygenas and Cytochrome P450 Reductase Candidates

Using a standard lithium acetate protocol, the recombinant plasmid pTIaAO2 was transformed into *S. cerevisiae* WAT11tfAX, which harbors *IaAS*1 and can synthesize α- and β-amyrin efficiently (Supplementary Table 2). The resulted strain was named as WAT11tfAX-pT*IaAO2*. Accordingly, pT*IaAO2*-*IaCPR* was separately transferred into strain WAT11tfAX to give the strain WAT11tfAX-pT*IaAO2*-Ia*CPR*. The plasmids pT*IaAO1* (Ji et al., 2020) and pU*IaAO4* were co-transformed into *S. cerevisiae* WAT11tfAX. The resulted strain was named as WAT11tfAX-pT*IaAO1*-pU*IaAO4*. In addition, strain WAT11tfAX-pT*IaAO1* which contained only plasmid pT*IaAO1* and strain WAT11tfAX-pT*IaAO1*-pU which contained plasmid pT*IaAO1* and empty vector pESC-URA were constructed and used as negative control. On the other hand, the plasmids pT*IaAO5* mentioned above and pYES-DEST52 *IaAS2* carrying the amyrin synthase *IaAS2* from *I. asprella* (Zheng et al., 2015) were co-transformed into the engineered strain of *S. cerevisiae* WAT11tfA (Supplementary Table 2) to give strain WAT11tfA-pD*IaAS2*-pT*IaAO5*. For negative control, strain WAT11tfA-pD*IaAS2* and strain WAT11tfA-pD*IaAS2*-pT were used. The expression of recombinant protein was determined by western blotting. All the engineered yeast strains were first grown in 15 ml culture tubes containing 5 ml SC-Dropout medium with 20 g/L glucose at 30°C and 225 rpm for 48-h. And then the strains were washed three times with sterile water, re-suspended in SC-Dropout medium with 20 g/L galactose and further grown at 30°C and 225 rpm for 7 days.

### Extraction and Analysis of Metabolites From Recombinant Yeast

Yeast cells incubated in induction media for 7 days were collected, refluxed in 20% KOH/50% ethanol for 30 min, and then extracted with hexane. Extracts were evaporated to dryness, silylanized with BSTFA reagent (30 min at 80°C) (Sigma, Shanghai, China), then re-suspended in hexane and submitted to gas chromatography mass spectrometry (GC-MS) analysis. GC-MS analysis was performed on an Agilent 7890B GC machine equipped with an HP-5MS column (0.25 mm × 30 m × 0.25 μm, Agilent, Santa Clara, CA, United States). The column temperature was set at 150°C for 2 min, followed by a 40°C/min ramp to 300°C, held at 300°C for 20 min. Injector and detector temperatures were both set at 250°C. The sample was injected in a splitless mode. The carrier gas was helium with a flow rate of 1.2 mL/min. Ionization of samples was performed by electron impact at 70 eV, with a mass range acquired over *m/z* 50–600. The peaks were identified by matching retention times, fragmentation of mass spectra with authentic standards as well as comparison with NIST Mass Spectral Library.

### Subcellular Localization Analyses

To determine the subcellular localization of IaAO2, IaAO4, IaAO5, and IaCPR protein, the encoding region of four genes without the stop codon were fused in-frame to the N-terminal of Enhanced Green Fluorescent Protein (EGFP) *via* the *Spe*I - *Bam*HI sites in the vector pAN580, resulting in four plant expression constructs pAN580-IaAO2, pAN580-IaAO4, pAN580-IaAO5, and pAN580-IaCPR. They were transformed into *Arabidopsis* protoplasts by polyethylene glycol (PEG)-mediated transfection as described previously (Yoo et al., 2007). The plant transformed with EGFP empty vector was used as control. After transformation, the protoplasts were incubated at 28°C for 16 h and then examined using a confocal laser scanning microscope (Zeiss LSM 800, Germany). The excitation wavelengths for GFP was 488 nm.

### qPCR Analysis

Roots, stems and leaves were collected from well-growth, 1-year-old *I. asprella* plants with height between 40–60 cm. Total RNA was isolated and reverse-transcribed to cDNA by the 5 × All-In-One RT MasterMix (with AccuRT Genomic DNA Removal Kit) (ABM, Canada). qPCR was carried out using the TB Green *Premix Ex Taq* (Takara, Dalian, China). The conditions of two-step real-time PCR were 95°C for 30 s, followed by 40 cycles at 95°C for 5 s and 60°C for 30 s. The relative expression value of the selected genes was estimated using the ^–ΔΔ^CT method (Livak and Schmittgen, 2001). 18S rRNA gene was used for normalization. Three biological replicates were set up in each group. The primers designed for this study are listed in Supplementary Table 1.

### Construction of Engineered *Saccharomyces cerevisiae via* CRISPR/Cas9

A yeast strain carrying *IaAO*1, *IaAO*4, and *IaCPR* genes was constructed. *IaAO*1, *IaAO*4 and *IaCPR* were sub-cloned into the yeast expression vector p426GPD to create the expression cassettes *P*_*GAP*_-*IaAO1*-*T*_*CYC*1_, *P*_*GAP*_-*IaAO4*-*T*_*CYC*1_ and *P*_*GAP*_-*IaCPR*-*T*_*CYC*1_, respectively. The *P*_*GAP*_-*IaAO1*-*T*_*CYC*1_ cassette was integrated into the *leu2* locus of strain WAT11tfAX *via* CRISPR/Cas9, then *P*_*GAP*_-*Ia*AO4-*T*_*CYC*1_ and *P*_*GAP*_-*IaCPR*-*T*_*CYC*1_ were subsequently integrated into the *ura3* and *ade2* locus, resulting in strain WAT11L. The constructed yeast strain was cultured in YPD medium at 30°C for 10-day. After that, yeast metabolites were extracted by alkaline lysis and used for GC-MS detection, as described above (Ji et al., 2020).

## Results

### Candidate Cytochrome P450 Monooxygenas and Cytochrome P450 Reductase Genes Involved in Triterpenoid Biosynthesis

**FIGURE 2:**
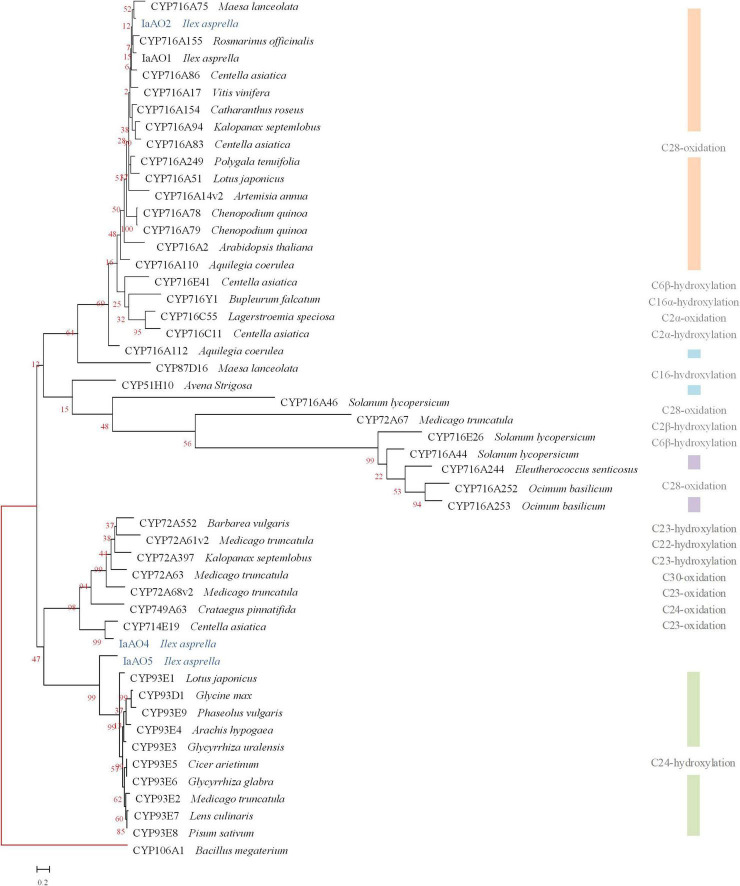
Phylogenetic analysis of IaAO2, IaAO4, and IaAO5 with known triterpene CYPs. The relevant gene names and amino acid sequences are given in Supplementary Material. Genes isolated in this study were colored in blue. Red branches represented outgroup. Scale bar indicated the number of amino acid substitutions.

It is reported that the amino acid sequence homology between CYPs belonging to the same family should be more than 40%, and the same subfamily should be more than 55% (Nelson, 2009). Sequence alignment and phylogenetic analysis revealed three *CYP* genes potentially involved in oxidative modification in triterpenoid biosynthesis, namely *IaAO*2, *IaAO*4, and *IaAO*5 (Figure 2).

Among them, gene *IaAO2* (a CYP716A212 homolog, GenBank accession No. OL604227) is 1,239 bp in length, encoding a protein of 412 aa. TMHMM Server v2.0 online tool was used to predict the transmembrane domain of IaAO2 protein. The results showed that IaAO2 protein was located outside the cell membrane and there was no transmembrane domain (Supplementary Figure 4). IaAO2 was closely clustered with CYP716A75, which was identified from *Maesa lanceolata* as a multifunctional C-28 oxidase capable of converting β-amyrin and 16α-hydroxy β-amyrin to oleanolic acid and 16α-hydroxy oleanolic acid, respectively (Moses et al., 2015). The sequence similarity between IaAO2 and CYP716A75 is 60%. It is worth mentioning that the N-terminal of IaAO2 was 66 amino acids less than IaAO1, but its functional domain structure was complete (Supplementary Figure 4). The similarity of amino acid sequences encoded by IaAO2 and IaAO1 is 76.46%. Therefore, it is plausible that *IaAO2* encodes a triterpene C-28 oxidase.

Sequence analysis of *IaAO4* (CYP714E88, GenBank accession No. MZ508437) revealed an open reading frame of 1,539 bp, encoding a protein of 513 aa. IaAO4 was closely clustered with CYP714E19, which was identified from *Centella asiatica* as a multifunctional C-23 oxidase capable of converting ursolic acid and oleanolic acid to 23-carboxyl-ursolic acid and gypsogenin in three successive steps, respectively (Kim et al., 2018). The sequence similarity between IaAO4 and CYP714E19 is 63.32%. Thus IaAO4 is very likely a triterpene C-23 oxidase.

The other putative CYP gene *IaAO5* (CYP93A220, GenBank accession No. MZ508433) includes an open reading frame of 1,545 bp, encoding a protein of 515 aa. IaAO5 was closely clustered with CYP93E1, which was identified from *Glycine max* as a C-24 hydroxylase capable of converting β-amyrin to olean-12-ene-3β, 24-diol (Shibuya et al., 2006; Moses et al., 2014). The sequence similarity between IaAO5 and CYP93E1 is 49.42%. Therefore, IaAO5 might be a triterpene C-24 hydroxylase, too.

**FIGURE 3:**
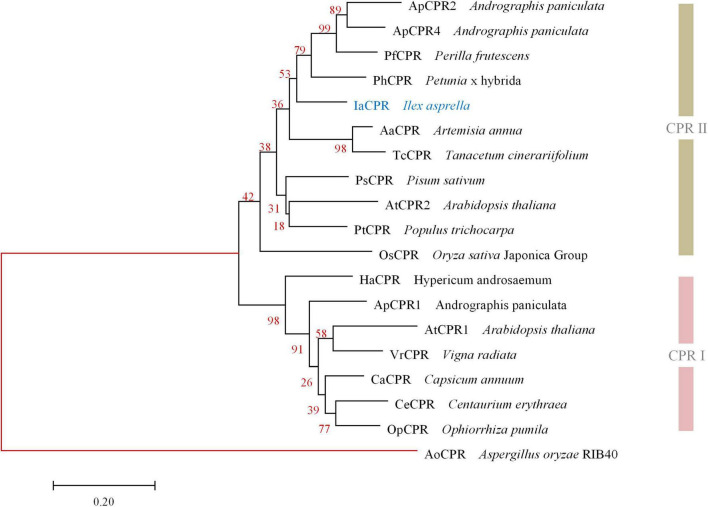
Phylogenetic analysis of IaCPR with known CPRs. The gene names and sequences are given in Supplementary Material. Genes isolated in this study were colored in blue. Red branches represented outgroup.

Likewise, *IaCPR* (GenBank accession No. MZ508433) annotated as “cytochrome P450 reductase” contains an open reading frame of 2,133 bp, encoding a 710 aa protein. According to phylogenetic tree analysis, IaCPR was divided into CPR II family (Figure 3).

By using RNA from the leaves of *I. asprella* as template, cDNA of *IaAO2*, *IaAO4*, *IaAO5* and *IaCPR* were obtained.

### IaCPR Acts as NADPH-Cytochrome P450 Reductase

The recombinant plasmid pET32a*-IaCPR* was successfully constructed and transformed into *E. coli* Transetta (DE3) to induce the expression of IaCPR. After purification of the target protein, SDS-PAGE analysis showed that the size of the recombinant protein was consistent with the expectation (IaCPR: 89.3 kDa), indicating that IaCPR was successfully expressed. Its enzymatic properties were analyzed (Supplementary Figure 2). The *in vitro* assays showed that IaCPR could reduce cytochrome c and K_3_Fe(CN)_6_ in a NADPH dependent manner, indicating it has the function of CPR (Supplementary Figure 2). Under these reaction conditions, the kinetic parameter *Vmax* of IaCPR for substrate cytochrome c was calculated as 3.86 μmol/min/mg, *Km* was 21.55 μmol/L. The kinetic parameter *Vmax* of IaCPR for substrate NADPH was 2.56 μmol/min/mg, *Km* was 7.69 μmol/L. The kinetic parameter *Vmax* of IaCPR for substrate K_3_Fe(CN)_6_ was 6.75 μmol/min/mg, *Km* was 14.79 μmol/L.

### Cytochrome P450 Monooxygenas Candidates Catalyze Regio-Specific Oxidation of Pentacyclic Triterpenes

To identify the functions of *CYP* candidate genes, two previously engineered yeast strains WAT11tfAX and WAT11tfA were engaged. WAT11tfA accumulates the common triterpene precursor 2, 3-oxidosqualene, while WAT11tfAX accumulates the triterpenoid intermediates α- and β-amyrin.

**FIGURE 4:**
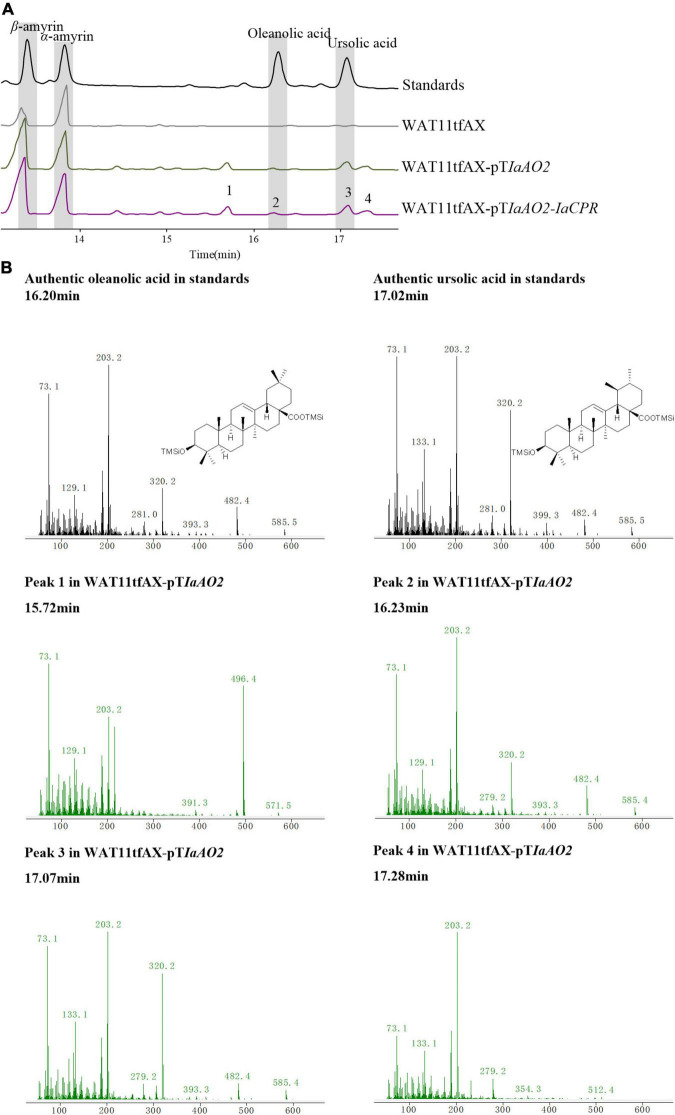
GC-MS analysis of metabolites in WAT11tfAX-pT*IaAO2*. **(A)** Total ion chromatograms of mixed standard (black line), WAT11tfAX (gray line), WAT11tfAX-pT*IaAO2* (green line), and WAT11tfAX-pT*IaAO1*-*IaCYP* (purple line). Major peaks were numbered. **(B)** Mass spectrum of ursolic acid, oleanolic acid and peak 1–4 from the GC profile shown in panel **(A)**. The retention time and mass spectra of peak 2 compared well with those of oleanolic acid. The retention time and mass spectra of peak 3 compared well with those of ursolic acid. The chemical structure shown were TMS-derivatized. GC-MS analysis was performed with a HP-5MS column.

In case of *IaAO2*, it was hetero-expressed separately or together with IaCPR in yeast strain WAT11tfAX. Western blot analysis showed that IaAO2 was expressed as expected (Supplementary Figure 1). The metabolites of strains WAT11tfAX-pT*IaAO2* and WAT11tfAX-pT*IaAO2-IaCPR* after 7 days of cultivation were analyzed, using WAT11tfAX as a negative control. WAT11tfAX-pT*IaAO2* accumulated four additional peaks (peak 1∼4, Figure 4A). Peak 2 and 3 were determined as oleanolic acid (OA) and ursolic acid (UA), respectively (Figure 4B). Peaks 1 and 4 were preliminarily confirmed as uvaol (matching degree 94%) and ursolic aldehyde (matching degree 99%) through NIST14/Wiley275 Mass Spectral Library. And the control strain did not accumulate these products. The results demonstrated that IaAO2 is a C-28 triterpene oxidase, catalyzing the oxidation of α- and β-amyrin to OA and UA, respectively. Furthermore, strain WAT11tfAX-pTIaAO2-IaCPR also accumulated OA and UA but in larger quantities (964.73 μg/L UA, about 2.5-fold as that of WAT11tfAX-pT*IaAO2*), suggesting that IaCPR could promote the catalytic efficiency of IaAO2 in *S. cerevisiae* (Table 1).

**TABLE 1:** Accumulation of substrates and products in yeast strains expressing *IaAO2*.

Yeast strain	Accumulation of substrate and products (μg/L)			
	β-amyrin	α-amyrin	oleanolic acid	ursolic acid
WAT11tfAX-pT*IaAO2*	28.08	153.89	27.53	382.82
WAT11tfAX-pT*IaAO2-IaCPR*	102.18	649.78	25.16	964.73

**FIGURE 5:**
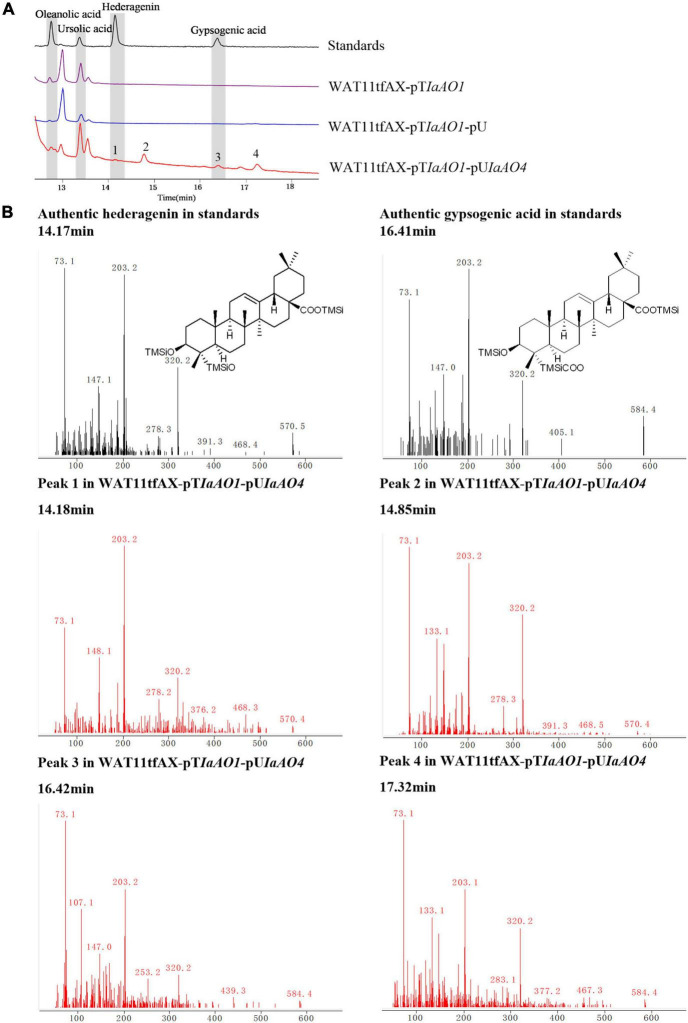
GC-MS analysis of metabolites in WAT11tfAX-pT*IaAO1*-pU*IaAO4*. **(A)** Total ion chromatograms of mixed standard (black line), WAT11tfAX-pT*IaAO1* (purple line), WAT11tfAX-pT*IaAO1*-pU (blue line), and WAT11tfAX-pT*IaAO1*-pU*IaAO4* (red line). Major peaks were numbered. **(B)** Mass spectrum of hederagenin, gypsogenic acid and peak 1–4 from the GC profile shown in panel **(A)**. The retention time and mass spectra of peak 1 compared well with those of hederagenin. The retention time and mass spectra of peak 3 compared well with those of gypsogenic acid. In addition, the mass spectra of peak 2 was similar to that of hederagenin and the peak 4 was similar to that of gypsogenic acid.

To identify the function of *IaAO4*, it was co-expressed in the yeast strain WAT11tfAX together with IaAO1 for biochemical characterization and metabolite determination. Western blot analysis revealed the successful expression of IaAO4 (Supplementary Figure 1). As expected, the expression of IaAO1 in WAT11tfAX led to the production of OA and UA. The expression of pESC-URA did not lead to any additional products. In comparison, co-expression of IaAO1 and IaAO4 resulted in the formation of four new peaks (Figure 5A). Among the newly formed peaks, the peak 1 and 3 were identified as hederagenin and gypsogenic acid (GA), based on the consistence of retention times and mass spectra with those of authentic standards. Peak 2 and 4 displayed the characteristic fragment ions *m/z* 203 and 320 of pentacyclic triterpenes. In addition, peak 2 showed MS fragmentation pattern similar to that of hederagenin (Figure 5B) and peak 4 showed pattern similar to that of GA, implying peak 1 and 2, peak 3 and 4 must be two pairs of isomers. Therefore, peak 2 and 4 were tentatively identified as 23-hydroxyl-ursolic acid and 23-carboxyl-ursolic acid. These results demonstrate that IaAO4 is a multifunctional triterpenoid oxidase, catalyzing the oxidation of both OA and UA at the C-23 position, most likely in three successive steps.

**FIGURE 6:**
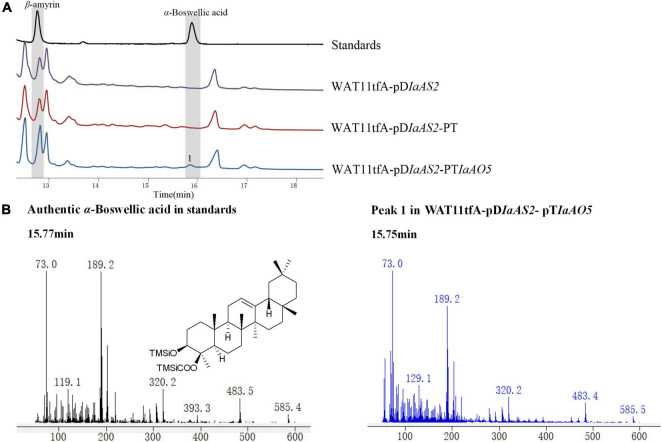
GC-MS analysis of metabolites in WAT11tfA-pD*IaAS2*-pT*IaAO5*. **(A)** Total ion chromatograms of mixed standard (black line), WAT11tfA-pD*IaAS2* (purple line), WAT11tfA-pD*IaAS2*-pT (red line), and WAT11tfA-pD*IaAS2*-pT*IaAO5* (blue line). Major peaks were numbered. **(B)** Mass spectrum of α-Boswellic acid and peak 1 from the GC profile shown in panel **(A)**. The retention time and mass spectra of peak 1 compared well with those of α-Boswellic acid.

**FIGURE 7:**
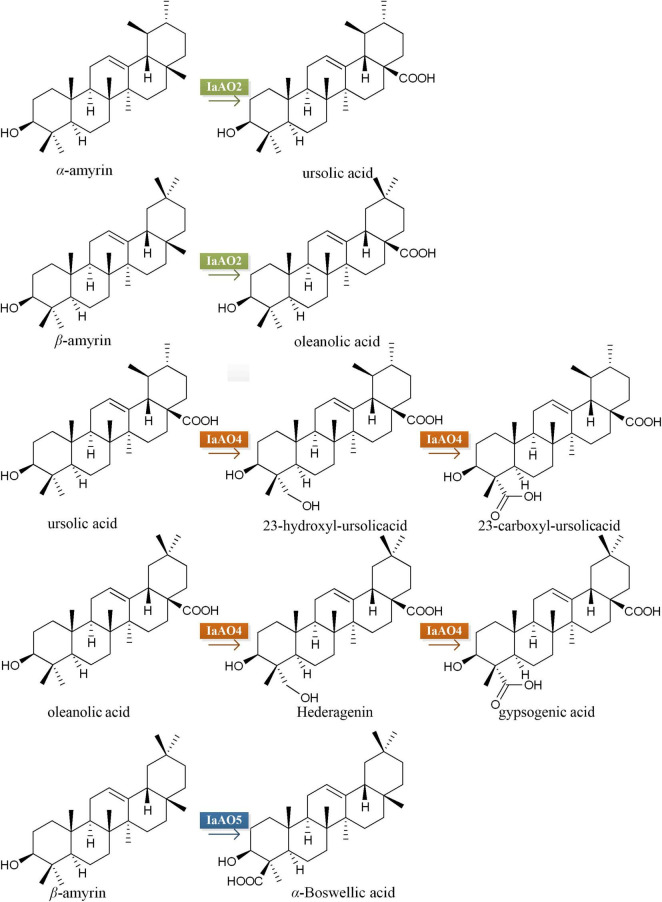
Speculation on biosynthetic pathway of IaAO2, IaAO4, and IaAO5 in engineered yeast.

For functional characterization of IaAO5, it was firstly carried out by using α- and β-amyrin producing strain WAT11tfAX. However, no newly formed product was detected. Therefore, yeast strain WAT11tfA-pD*IaAS2*, WAT11tfA-pD*IaAS2*-pT and WAT11tfA-pD*IaAS2*-pT*IaAO5* were constructed. Western blot analysis revealed IaAO5 was successfully expressed in yeast (Supplementary Figure 1). GC-MS analysis showed WAT11tfA-pDIaAS2 produced β-amyrin as anticipated and WAT11tfA-pD*IaAS2*-pT accumulated no new compounds other than β-amyrin (Figure 6A). In contrast, the WAT11tfA-pD*IaAS2*-pT*IaAO5* produced one additional compound which was not found in the controls. The compound was identified as α-Boswellic acid after compared with the authentic standard in regard of retention time and mass spectra (Figure 6B). These results demonstrate that IaAO5 from *I. asprella* catalyzes oxidation at the C-24 position of β-amyrin to yield α-Boswellic acid (Figure 7).

### Tissue-Specific Expression and Subcellular Localization

**FIGURE 8:**
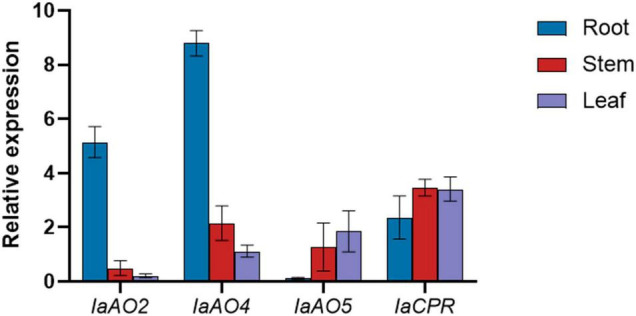
qPCR analysis of *CYPs* and *CPR* in different parts of *I. asprella* plants. The relative fold expression of genes in different organs (root, stem, and leaf) is shown.

Gene expression determined by qPCR analysis revealed that the expression level of *IaAO2* mRNA was highest in roots. *IaAO4* showed a similar mRNA accumulation pattern as *IaAO2*. The mRNA of the CYP gene *IaAO5* was expressed at slightly higher levels in leaves. Different from CYPs, *IaCPR* showed similar expression levels in roots, stems and leaves (Figure 8).

The TMHMM Server v2.0 have shown that IaAO4, IaAO5, and IaCPR are all transmembrane proteins, while IaAO2 lack of transmembrane domain. The results of subcelluer localization using the Arabidopsis protoplast system were consistent with the prediction. In the case of *IaAO4*, *IaAO5* and *IaCPR*, it was found that the GFP fluorescence upon microscopic examination overlapped with chlorophyll emission fluorescence. It is speculated that they locate on the chloroplast membrane. On the contrary, the fluorescence signals of protoplasts transformed with IaAO2 were distributed in cytoplasm and plast, revealing that IaAO2 was located outside the cell membrane (Supplementary Figure 4).

### Construction of the Biosynthetic Pathway of Pentacyclic Triterpenoid Derivatives in Yeast

**FIGURE 9:**
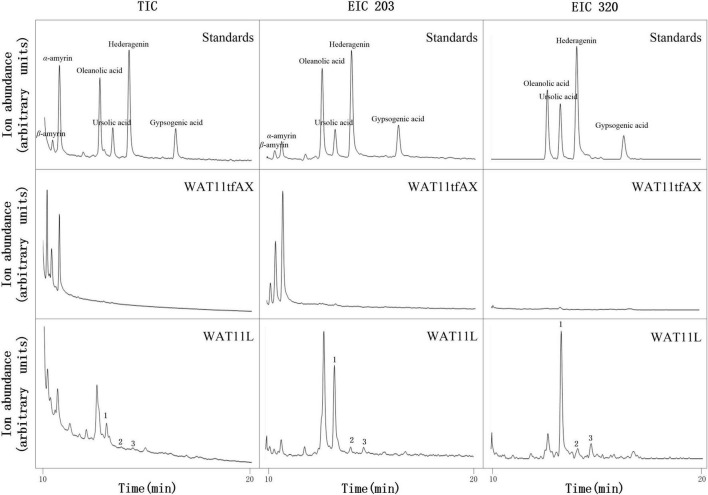
GC-MS analysis of metabolites in WAT11L. The total ion flow diagram of mixed standard, control yeast strain WAT11tfAX and WAT11tfAX-derived strain WAT11L were marked separately in the figure. In TIC mode analysis, compared with WAT11tfAX, WAT11L generated three new peaks 1, 2, and 3. The retention time of peak 1 and 2 in WAT11L compared well with those in mixed standard. In single ion mode (*m/z* 203, 320), the peak of 1, 2, and 3 were more obvious. In addition, the GC retention time difference between peaks 2 and 3 of the two compounds matched the retention time difference between ursolic acid and oleanolic acid.

**FIGURE 10:**
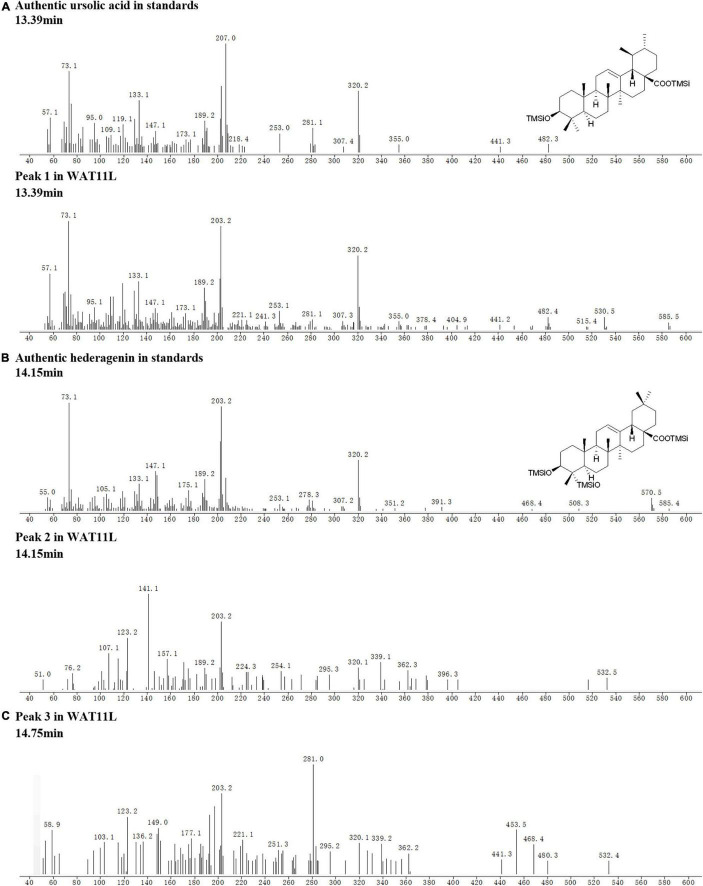
Tentative MS identification of triterpenoids from WAT11L. **(A)** Compound 1 was tentatively identified as ursolic acid. **(B)** Compound 2 was tentatively identified as hederagenin. **(C)** The figure showed the total ion flow diagram of compound 3.

Based on  the *CYP*s isolated from *I. aprella*, they were inserted into the genome of yeast strain WAT11tfAX  with the aim to obtain strains  producing pentacyclic triterpenoid derivatives. Genes *IaAO*4 and *IaAO*1 were firstly optimized according to the codon usage of *S. cerevisiae* and then integrated into the yeast genome *via* CRISPR/Cas9 technology, resulting in strain WAT11L. After 10-day culture, the metabolites of engineered yeast strains were submitted to GC-MS analysis. The results showed that WAT11L produced some new products that were not found in the control strain WAT11tfAX. By using Total Ion Counts (TICs) mode and Extracted Ion Chromatograms (EICs) mode, three peaks (peak 1, 2, and 3) were detected unequivocally. Peak 1 was identified as UA, as its GC retention time (Figure 9) and MS fragmentation pattern (Figure 10A) was identical to authentic standard UA. Peak 2 was tentatively identified as hederagenin based on its retention time (Figure 9) and MS fragmentation pattern (Figure 10B) similar to authentic standard hederagenin. The MS fragmentation pattern of peak 3 resembled that of peak 2 (Figure 10C), implying it might be the isomer of peak 2, 23-hydroxyl-ursolic acid. However, its exact structures could not be assigned based on the evidence acquired here. What’s more, it was worthy to mention that in both TICs and EICs mode (*m/z* 203, 320) there were still many other compounds that can be detected. The strain WAT11L must accumulate several triterpenoid derivatives including C-23 hydroxylation triterpenoids.

## Discussion

The vast structural diversity of plant triterpenoids is largely created by CYPs, the key enzymes from the downstream biosynthetic pathways. In this study, we identified three CYPs from *I. asprella*, namely IaAO2, IaAO4, and IaAO5. They are responsible of various oxidative tailoring reactions in the biosynthesis of ursane- and oleanane-type triterpenoids. IaAO2 could convert α- and β-amyrin into UA and OA, respectively. This is the second C-28 oxidase from *I. asprella*. According to previous report, 90% of triterpenoids in *I. asprella* are oxidized at C-28 position (Ji et al., 2020). Therefore, it can be inferred that C-28 oxidase is the key enzyme catalyzing the initial step of multiple oxidation of triterpenoids. Notably, IaAO2 contains no transmembrane domain, unlike other characterized CYPs. And the lack of transmembrane domain did not affect its catalytic activity in heterologous host yeast. Quantitative PCR revealed the unambigous expression of IaAO2 in different tissues of *I. asprella*, suggesting it must play a role in plant. However, its exact function is unknown and need to be further elucidated.

Another CYP identified in this study IaAO4, was shown to be a multifunctional triterpenoid C-23 oxidase, accepting both OA and UA. A similar enzyme CYP714E19 has been isolated from *Centella asiatica* (Kim et al., 2018). According to the author, CYP714E19 plays a major role in oxidizing the C-23 methyl group of various triterpenoid precursors into corresponding 23-hydroxy compounds, and only a lesser role in the two further oxidation steps to the corresponding aldehydes and acids. In comparison, IaAO4 catalyzed the oxidation of UA and OA to the corresponding alcohols and acids, no aldehyde products at C-23 position can be detected. Besides, there are two more C-23 oxidases, CYP72A397 and CYP72A552, which were proved to accept only oleanane-type substrates (Seki et al., 2011; Han et al., 2018; Liu et al., 2019; Tzin et al., 2019). Investigation into the interactions between these CYPs and substrates may shed light on the reasons for differences in their substrate specificity profile.

The third CYP, IaAO5 catalyzes the carboxylation at the C-24 position of β-amyrin to yield α-Boswellic acid. The catalytic reaction was observed only in β-amyrin producing strain WAT11tfA-pD*IaAS2* expressing *IaAO*5 but not in α- and β-amyrin producing strain WAT11tfAX containing *IaAO*5. The reasons may lie in low amount of the substrate β-amyrin in the strain WAT11tfAX and poor catalytic efficiency of IaAO5. So far, several triterpenoid C-24 oxidases have been reported, but their functions seem vary slightly from one another. CYP749A63 catalyzes C-24 hydroxylatin of OA. CYP93E subfamily members like CYP93E1 and CYP93E2 convert β-amyrin to the product olean-12-ene-3β, 24-diol (Shibuya et al., 2006; Moses et al., 2014; Dai et al., 2019). Compared to that, IaAO5 from *I. asprella* must catalyze the three-step oxidation of methyl group of β-amyrin, though no corresponding alcohol and aldehyde at C-24 has been detected in our study.

Versatile triterpenoids and triterpenoid saponins have been isolated *asprella*, most of which are oxidized at various positions. The isolation of different CYPs from *I. asprella* is not only consistent with multiple oxidative modification of triterpenoids, but also provides valuable synthetic biology tools for metabolic engineering. Yeast strain WAT11L with the integration of three genes IaCPR, IaAO1 and IaAO4 by CRISPER/Cas9 produces probably C-23 hydroxylation triterpenes, as expected. Yeast strain WAT11tfAX harboring the gene IaAO2 accumulated UA and OA and subsequent introducing another copy of CYP redox partner, IaCPR, increased the production up to 2.5-fold. In conclusion, the characterization of IaAO2, IaAO4, IaAO5, and IaCPR may give rise to new potential approaches for genetic engineering to enhance large-scale production of biological active triterpenoid saponins of *I. asprella* and provide precursor substrates for the identification of more new oxidases.

## Data Availability Statement

The datasets presented in this study can be found in online repositories. The names of the repository/repositories and accession number(s) can be found in the article/Supplementary Material.

## Author Contributions

HX and LL designed the experiment and wrote the manuscript. LL, SL, and YC performed the major experiments including gene cloning, enzyme expression, and functional characterization. YW and LX contributed to the GC-MS analysis. LS and XY provided the helpful guidance. HX and RZ supervised the entire project. All authors read and approved the manuscript.

## Conflict of Interest

The authors declare that the research was conducted in the absence of any commercial or financial relationships that could be construed as a potential conflict of interest.

## Publisher’s Note

All claims expressed in this article are solely those of the authors and do not necessarily represent those of their affiliated organizations, or those of the publisher, the editors and the reviewers. Any product that may be evaluated in this article, or claim that may be made by its manufacturer, is not guaranteed or endorsed by the publisher.

## Footnotes

1
https://www.ncbi.nlm.nih.gov/nuccore/

2
http://www.cbs.dtu.dk/services/TMHMM/

## References

[B1] Bao T. Ke Y. Wang Y. Wang W. Li Y. Wang Y. (2018). Taraxasterol suppresses the growth of human liver cancer by upregulating Hint1 expression. *J. Mol. Med. (Berl)* 96 661–672. 10.1007/s00109-018-1652-7 29806073

[B2] Dai Z. Liu Y. Sun Z. Wang D. Qu G. Ma X. (2019). Identification of a novel cytochrome P450 enzyme that catalyzes the C-2α hydroxylation of pentacyclic triterpenoids and its application in yeast cell factories. *Metab. Eng.* 51 70–78. 10.1016/j.ymben.2018.10.001 30339834

[B3] Du B. Zhao F. Zhang H. Feng X. Xing J. Han Z. (2018). Asprenols A-H, phenolic constituents from the stems of Ilex asprella. *Fitoterapia* 129 220–225. 30031114 10.1016/j.fitote.2018.07.011

[B4] Felsenstein J. (1985). Confidence limits on phylogenies: an approach using the bootstrap. *Evolution* 39 783–791. 10.1111/j.1558-5646.1985.tb00420.x 28561359

[B5] Fukushima E. O. Seki H. Sawai S. Suzuki M. Ohyama K. Saito K. (2013). Combinatorial biosynthesis of legume natural and rare triterpenoids in engineered yeast. *Plant Cell Physiol.* 54 740–749. 10.1093/pcp/pct015 23378447

[B6] Guengerich F. P. Martin M. V. Sohl C. D. Cheng Q. (2009). Measurement of cytochrome P450 and NADPH-cytochrome P450 reductase. *Nat. Protoc.* 4 1245–1251. 10.1038/nprot.2009.121 19661994 PMC3843963

[B7] Han J. Y. Chun J. H. Oh S. A. Park S. B. Hwang H. S. Lee H. (2018). Transcriptomic analysis of *Kalopanax septemlobus* and characterization of KsBAS, CYP716A94 and CYP72A397 genes involved in hederagenin saponin biosynthesis. *Plant Cell Physiol.* 59 319–330. 10.1093/pcp/pcx188 29186583

[B8] Han J. Y. Kim M. J. Ban Y. W. Hwang H. S. Choi Y. E. (2013). The involvement of β-amyrin 28-oxidase (CYP716A52v2) in oleanane-type ginsenoside biosynthesis in Panax ginseng. *Plant Cell Physiol.* 54 2034–2046. 24092881 10.1093/pcp/pct141

[B9] Huang X. J. Wen S. Guan X. F. Wu Z. L. Li M. M. Fan C. L. (2019). Eleven new triterpenoid glycosides from the roots of ilex asprella. *Chem. Biodivers.* 16:e1900202. 10.1002/cbdv.201900202 31115136

[B10] Ji X. Lin S. Chen Y. Liu J. Yun X. Wang T. (2020). Identification of α-Amyrin 28-Carboxylase and Glycosyltransferase From Ilex asprella and Production of Ursolic Acid 28-O-β-D-Glucopyranoside in engineered yeast. *Front. Plant Sci.* 11:612. 10.3389/fpls.2020.00612 32508864 PMC7251064

[B11] Kim O. T. Um Y. Jin M. L. Kim J. U. Hegebarth D. Busta L. (2018). A novel Multifunctional C-23 Oxidase, CYP714E19, is involved in asiaticoside biosynthesis. *Plant Cell Physiol.* 59 1200–1213. 10.1093/pcp/pcy055 29579306

[B12] Kumar S. Stecher G. Tamura K. (2016). MEGA7: molecular evolutionary genetics analysis version 7.0 for bigger datasets. *Mol. Biol. Evol.* 33 1870–1874. 10.1093/molbev/msw054 27004904 PMC8210823

[B13] Li L. He Y. X. Gou M. L. Dai C. (2012). Three new triterpenoid saponins from *Ilex pubescens*. *J. Asian Nat. Prod. Res.* 14 1169–1174. 23134324 10.1080/10286020.2012.738674

[B14] Liby K. T. Yore M. M. Sporn M. B. (2007). Triterpenoids and rexinoids as multifunctional agents for the prevention and treatment of cancer. *Nat. Rev. Cancer* 7 357–369. 10.1038/nrc2129 17446857

[B15] Liu Q. Khakimov B. Cárdenas P. D. Cozzi F. Olsen C. E. Jensen K. R. (2019). The cytochrome P450 CYP72A552 iskey toproductionof hederagenin-based saponins that mediate plant defense against herbivores. *New Phytol.* 222 1599–1609. 10.1111/nph.15689 30661245

[B16] Liu X. Zhu X. Wang H. Liu T. Cheng J. Jiang H. (2020). Discovery and modification of cytochrome P450 for plant natural products biosynthesis. *Synth. Syst. Biotechnol.* 5 187–199. 10.1016/j.synbio.2020.06.008 32637672 PMC7332504

[B17] Livak K. J. Schmittgen T. D. (2001). Analysis of relative gene expression data using real-time quantitative PCR and the 2^–ΔΔ^CT method. *Methods* 25 402–408. 10.1006/meth.2001.1262 11846609

[B18] Lou H. Li H. Zhang S. Lu H. Chen Q. (2021). A review on preparation of betulinic acid and its biological activities. *Molecules* 26:5583. 10.3390/molecules26185583 34577056 PMC8468263

[B19] Moses T. Pollier J. Faizal A. Apers S. Pieters L. Thevelein J. M. (2015). Unraveling the triterpenoid saponin biosynthesis of the African shrub *Maesa lanceolata*. *Mol. Plant* 8 122–135. 10.1016/j.molp.2014.11.004 25578277

[B20] Moses T. Thevelein J. M. Goossens A. Pollier J. (2014). Comparative analysis of CYP93E proteins for improved microbial synthesis of plant triterpenoids. *Phytochemistry* 108 47–56. 25453910 10.1016/j.phytochem.2014.10.002

[B21] Nazaruk J. Borzym-Kluczyk M. (2015). The role of triterpenes in the management of diabetes mellitus and its complications. *Phytochem. Rev.* 14 675–690. 10.1007/s11101-014-9369-x 26213526 PMC4513225

[B22] Nelson D. R. (2009). The cytochrome p450 homepage. *Hum. Genomics*, 4 59–65. 10.1186/1479-7364-4-1-59 19951895 PMC3500189

[B23] Ohnishi T. Szatmari A. M. Watanabe B. Fujita S. Bancos S. Koncz C. (2006). C-23 hydroxylation by Arabidopsis CYP90C1 and CYP90D1 reveals a novel shortcut in brassinosteroid biosynthesis. *Plant Cell* 18 3275–3288. 10.1105/tpc.106.045443 17138693 PMC1693957

[B24] Rana S. Lattoo S. K. Dhar N. Razdan S. Bhat W. W. Dhar R. S. (2013). NADPH-cytochrome P450 reductase: molecular cloning and functional characterization of two paralogs from Withania somnifera (L.) dunal. *PLoS One* 8:e57068. 10.1371/journal.pone.0057068 23437311 PMC3578826

[B25] Rasool S. Mohamed R. (2016). Plant cytochrome P450s: nomenclature and involvement in natural product biosynthesis. *Protoplasma* 253 1197–1209. 10.1007/s00709-015-0884-4 26364028

[B26] Seki H. Ohyama K. Sawai S. Mizutani M. Ohnishi T. Sudo H. (2008). Licorice beta-amyrin 11-oxidase, a cytochrome P450 with a key role in the biosynthesis of the triterpene sweetener glycyrrhizin. *Proc. Natl. Acad. Sci. U. S. A.* 105 14204–14209. 10.1073/pnas.0803876105 18779566 PMC2532699

[B27] Seki H. Sawai S. Ohyama K. Mizutani M. Ohnishi T. Sudo H. (2011). Triterpene functional genomics in licorice for identification of CYP72A154 involved in the biosynthesis of glycyrrhizin. *Plant Cell* 23 4112–4123. 10.1105/tpc.110.082685 22128119 PMC3246328

[B28] Seki H. Tamura K. Muranaka T. (2015). P450s and UGTs: key players in the structural diversity of triterpenoid saponins. *Plant Cell Physiol.* 56 1463–1471. 10.1093/pcp/pcv062 25951908 PMC7107090

[B29] Shibuya M. Hoshino M. Katsube Y. Hayashi H. Kushiro T. Ebizuka Y. (2006). Identification of beta-amyrin and sophoradiol 24-hydroxylase by expressed sequence tag mining and functional expression assay. *FEBS J.* 273 948–959. 10.1111/j.1742-4658.2006.05120.x 16478469

[B30] Taketa A. T. Gnoatto S. C. Gosmann G. Pires V. S. Schenkel E. P. Guillaume D. (2004). Triterpenoids from Brazilian Ilex species and their *in vitro* antitrypanosomal activity. *J. Nat. Prod.* 67 1697–1700. 15497942 10.1021/np040059+

[B31] Tamura K. Teranishi Y. Ueda S. Suzuki H. Kawano N. Yoshimatsu K. (2017). Cytochrome P450 Monooxygenase CYP716A141 is a Unique β-Amyrin C-16β Oxidase Involved in Triterpenoid Saponin Biosynthesis in Platycodon grandiflorus. *Plant Cell Physiol.* 58 874–884. 10.1093/pcp/pcx043 28371833

[B32] Tzin V. Snyder J. H. Yang D. S. Huhman D. V. Watson B. S. Allen S. N. (2019). Integrated metabolomics identifies CYP72A67 and CYP72A68 oxidases in the biosynthesis of *Medicago truncatula* oleanate sapogenins. *Metabolomics* 15:85. 10.1007/s11306-019-1542-1 31144047

[B33] Waterhouse A. M. Procter J. B. Martin D. M. A. Clamp M. Barton G. J. (2009). Jalview version 2–a multiple sequence alignment editor and analysis workbench. *Bioinformatics* 25 1189–1191. 10.1093/bioinformatics/btp033 19151095 PMC2672624

[B34] Yang X. Gao X. Du B. Zhao F. Feng X. Zhang H. (2018). Ilex asprella aqueous extracts exert *in vivo* anti-inflammatory effects by regulating the NF-κB, JAK2/STAT3, and MAPK signaling pathways. *J. Ethnopharmacol.* 225 234–243. 10.1016/j.jep.2018.06.037 29981433

[B35] Yoo S. D. Cho Y. H. Sheen J. (2007). Arabidopsis mesophyll protoplasts: a versatile cell system for transient gene expression analysis. *Nat. Protoc.* 2 1565–1572. 10.1038/nprot.2007.199 17585298

[B36] Zheng X. Luo X. Ye G. Chen Y. Ji X. Wen L. (2015). Characterisation of two oxidosqualene cyclases responsible for triterpenoid biosynthesis in Ilex asprella. *Int. J. Mol. Sci.* 16 3564–3578. 10.3390/ijms16023564 25664861 PMC4346913

[B37] Zheng X. Xu H. Ma X. Zhan R. Chen W. (2014). Triterpenoid saponin biosynthetic pathway profiling and candidate gene mining of the Ilex asprella root using RNA-Seq. *Int. J. Mol. Sci.* 15 5970–5987. 10.3390/ijms15045970 24722569 PMC4013608

[B38] Zhou M. Xu M. Ma X. X. Zheng K. Yang K. Yang C. R. (2012). Antiviral triterpenoid saponins from the roots of Ilex asprella. *Planta Med.* 78 1702–1705. 10.1055/s-0032-1315209 22890543

[B39] Zhu M. Wang C. Sun W. Zhou A. Wang Y. Zhang G. (2018). Boosting 11-oxo-β-amyrin and glycyrrhetinic acid synthesis in Saccharomyces cerevisiae via pairing novel oxidation and reduction system from legume plants. *Metab. Eng.* 45 43–50. 10.1016/j.ymben.2017.11.009 29196123

