# Supplementary material for: Cytochrome P450 Monooxygenase/Cytochrome P450 Reductase Bi-Enzymatic System Isolated From Ilex asprella for Regio-Specific Oxidation of Pentacyclic Triterpenoids
Source: Front Plant Sci. 2022 Mar 24;13:831401. doi: 10.3389/fpls.2022.831401 (PMC9004391; doi:10.3389/fpls.2022.831401)
Supplement: Supplementary file 1 [file Data_Sheet_1.PDF]

Supplementary Material

1 Supplementary Data

Accession Numbers

Accession data used in the phylogenetic analyses: CYP716A155 (MK592859); CYP716A86 (KU878848); CYP716A17 (AB619803); CYP716A154 (JN565975); CYP716A94 (KT150521); CYP716A83 (KU878849); **IaAO2 (OL604227)**; CYP716A75 (KF318733); CYP716A14v2 (KF309251); CYP716A249 (KY385302.1); CYP716A51 (AB706297); CYP716A78 (KX343075); CYP716A79 (KX343076); CYP716A110 (KU878864); CYP716A112 (KU878865); CYP716A2 (LC106013); CYP716E41 (KU878851); CYP716C55 (MG708191); CYP716C11 (KU878852); CYP716AY1 (KC963423); CYP87D16 (KF318735); CYP716A46 (XM_004243858); CYP72A67 (DQ335780); CYP716E26 (XM_004241773); CYP716A44 (AK329870); CYP716A244 (KX354739); CYP716A252 (JQ958967); CYP716A253 (JQ958968); CYP51H10 (DQ680852); CYP72A397 (KT150517); CYP72A61v2 (AB558145); CYP72A552 (MH252571); CYP72A63 (AB558146); CYP72A68v2 (AB558150); CYP749A63 (MF596155); CYP714E19 (KT004520); CYP93E1 (LC414182); CYP93E3 (AB437320); CYP93D1 (AF135485); CYP93E9 (KF906540); CYP93E4 (KF906535); CYP93E5 (KF906536); CYP93E6 (KF906537); CYP93E8 (KF906539); CYP93E2 (DQ335790); CYP93E7 (KF906538); CYP106A1 (ADF38708); **IaAO4 (MZ508437)**; **IaAO5 (MZ508433)**.

AaCPR (ABM88789.1); ApCPR1 (AQT38168.1); ApCPR2 (AQT38169.1); ApCPR4 (AQT38171.1); AtCPR1 (NP_194183.1); AtCPR2 (CAA46815.1); CaCPR (ACF17649.1); CeCPR (AAS92623.1); HaCPR (AAS00459.1); OpCPR (BAC41516.1); OsCPR (XP_015650780.1); PfCPR (ADC94831.1); PhCPR (AAZ39649.1); PsCPR (AAC09468.2); PtCPR (XP_006381796.1); TcCPR (AGO03799.1); VrCPR (A47298); AoCPR (XP_001821060.1); **IaCPR (OL604229)**.

2 Supplementary Figures and Tables

2.1 Supplementary Figures

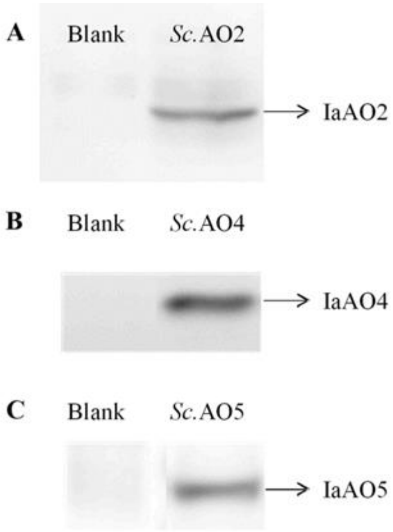

Supplementary Figure 1. Western blot analysis of IaAO2, IaAO4 and IaAO5 expressed in yeast. Empty vector containing yeast was indicated as blank.

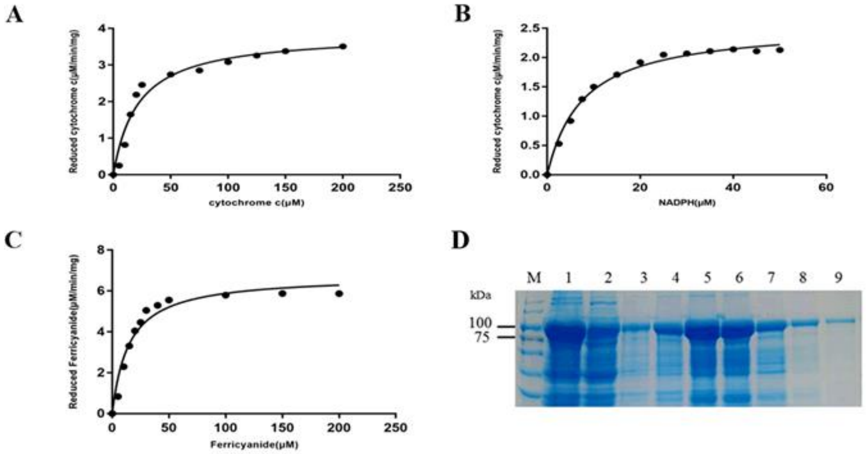

Supplementary Figure 2. Determination of in vitro enzyme activity of protein IaCPR. (A) Analysis of electron transfer activity of recombinant CPR on cytochromes c. (B) Analysis of electron transfer activity of recombinant CPR on NADPH. (C) Analysis of electron transfer activity of recombinant CPR on $K_3Fe(CN)_6$. (D) Expression and purification of target protein IaCPR. Marker was indicated as M. Total protein was indicated as 1. Supernatant protein was indicated as 2. Purified protein was indicated as 3-9.

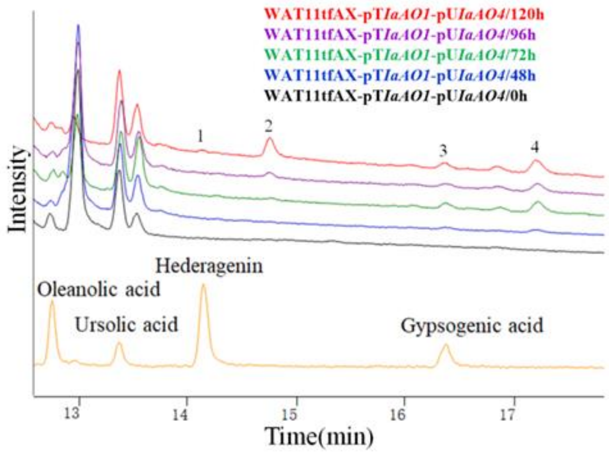

Supplementary Figure 3. GC-MS analysis of metabolites in WAT11tfAX-pTIaAO1-pUIaAO4 at different times when cultured in fermentor. Total ion chromatograms of mixed standard (orange line), WAT11tfAX-pUIaAO4-pTIaAO1 at 48-h (blue line), at 72-h (green line), at 96-h (purple line), at 120-h (red line), and blank control (black line) were shown and major peaks were numbered. The retention time and mass spectra of peak 1 and 3 compared well with those of hederagenin and gypsogenic acid. In addition, at 72-h, peak 3 and 4 were more obvious, while at 120-h, peaks 1-4 were more obvious. Therefore, it is speculated that the compound preferentially exists in the form of carboxylation when 23 position oxidation occurs. GC-MS analysis was performed with an HP-5MS column.

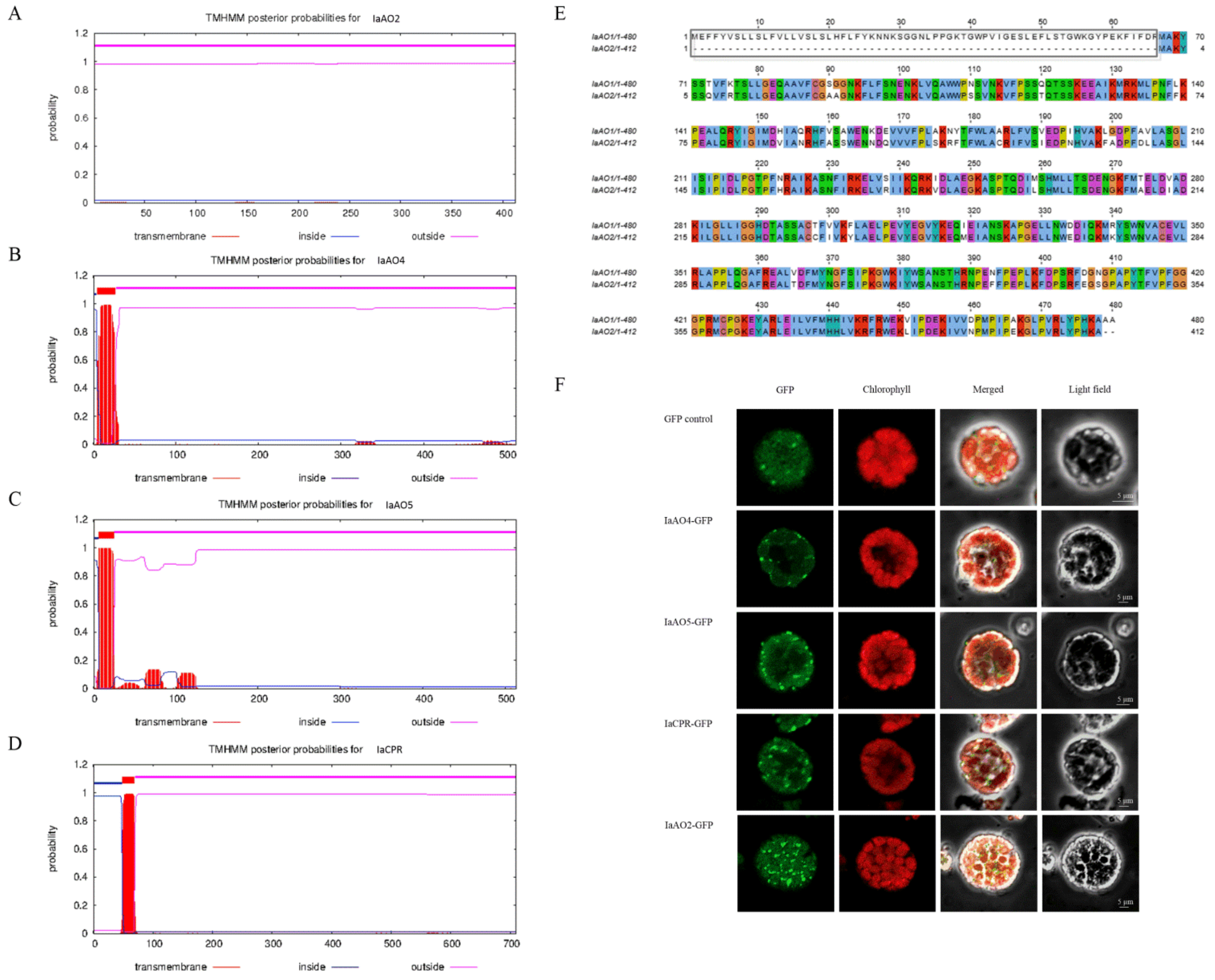

Supplementary Figure 4. Transmembrane domain prediction and subcellular localization of IaAO2, IaAO4, IaAO5 and IaCPR. **(A)** Transmembrane domain prediction of IaAO2. **(B)** Transmembrane domain prediction of IaAO4. **(C)** Transmembrane domain prediction of IaAO5. **(D)** Transmembrane domain prediction of IaCPR. **(E)** Multiple sequence alignment of IaAO1and IaAO2. **(F)** The subcellular localization of CYPs-GFP and CPR-GFP in *Arabidopsis* protoplasts was analyzed by confocal microscope. The GFP and chlorophyll were marked by green and red fluorescence. The light field was shown in white. The merged showed the localization of these CYPs.

2.2 Supplementary Tables

TABLE S1 Primers used in this study

Primer ID	(Primer sequence) 5’→3’	Remarks
IaCPR-F	ATGCAATCCAGCAACATCAAAGT	Amplification of *IaCPR*

IaCPR-R	TCACCACACGTCTCGCAGGTAC	
IaAO2-F	GGAGAAGTTCATCTTTGACC	Amplification of *IaAO2*
IaAO2-R	GCCTCTTATTATTACAGTGC	
IaAO4-F	ATGGAGGTCCAAGTTGTATTGA	Amplification of *IaAO4*
IaAO4-R	TTACAATTTCTTCACATAGAGATTGACC	
IaAO5-F	ATGGCCGACTTTCAAGGC	Amplification of *IaAO5*
IaAO5-R	CTATTTCAAAAGAAATGAATTGAGCCT	
V-pTIaCPR-F	GTAAGAATTTTTGAAAATTCGAATTCATGCAATCCAGCAACATCAAAGT	Amplification of *IaCPR* for ligation into pESC-TRP
V-pTIaCPR-R	CATCCTTGTAATCCATCGATACTAGTTCACCACACGTCTCGCAGGTA	
V-pTIaAO2-F	AAAAAACCCCGGATCCATGGCCAAATACTCTTCGCAAG	Amplification of *IaAO2* for ligation into pESC-TRP
V-pTIaAO2-R	ACCAAGCTTACTCGAGTTA***ATGATGATGATGATGATG***AGCTTTGTGAGGATAGAGGCGA	
pUIaAO4-F	TTGAAAATTCGAATTCATGGAGGTCCAAGTTGTATTGA	Amplification of *IaAO4* for ligation into pESC-URA
pUIaAO4-R	GAATTGTTAATTAAGAGCTCTTA***ATGATGATGATGATGATG***CAATTTCTTCACATAGAGATTGACC	
pTIaAO5-F	TTGAAAATTCGAATTCATGGCCGACTTTCAAGGC	Amplification of *IaAO5* for ligation into pESC-TRP
pTIaAO5-R	GAATTGTTAATTAAGAGCTCTAT***ATGATGATGATGATGATG***CTTCAAA	

	AGAAATGAATTGAGCCT	
32a-IaCPR-F	ACGACGACAAGGCCATGGCTGATATCATGAGAAGGTCGTTCGGACAAA	Amplification of IaCPR for ligation into pET32a
32a-IaCPR-R	CGGCCGCAAGCTTGTCGACGGAGCTCTCACCACACGTCTCGCAGGTA	
GPDIaAO1-F	TAGAACTAGTGGATCCATGGAGTTCTTCTATGTCTCTCTCCTCT	Amplification of *IaAO1* for ligation into p426GDP
GPDIaAO1-R	AATTACATGACTCGAGTTAAGCTGCTGCTTTGTGCGGA	
GPDIaAO4-F	TAGAACTAGTGGATCCATGGAAGTTCAAGTTGTTTTGAAAGTTTTGG	Amplification of *IaAO4* for ligation into p426GDP
GPDIaAO4-R	AATTACATGACTCGAGTTACAATTTTTTAACATACAAATTAACACCATTACCAGGTTCAA	
GPDIaCPR-F	TAGAACTAGTGGATCCATGCAATCCAGCAACATCAAAGTATCT	Amplification of *IaCPR* for ligation into p426GDP
GPDIaCPR-R	AATTACATGACTCGAGTCACCACACGTCTCGCAGGT	
ADE2-GAP-F	CATCCTACTATAACAATCAAGAAAAACAAGAAAATCGGACAAAACAATCAAGTGGGAACAAAAGCTGGAGCTCAGTTT	Amplification of P_{GAP}-*IaCPR*-T_{CYC1} expression cassette for insertion into ADE2 site of yeast genome
ADE2-CYC-R	GTATATCATTTTATAATTATTTGCTGTACAAGTATATCAATAAACTTATATAGGCCGCAAATTAAAGCCTTCGAGCGTCC	
LEU2-GAP-F	TTTACATTTCAGCAATATATATATATATATTTCAAGGATATACCATTCTAGGGAACAAAAGCTGGAGCTCAGTTT	Amplification of P_{GAP}-*IaAO1*-T_{CYC1} expression cassette for insertion into

LEU2-CYC-R	ATTTCATTTATAAAGTTTATGTACAAATATCATAAAAAAAGAGAATCTTTGGCCGCAAATTAAAGCCTTCGAGCGTCC	LEU2 site of yeast genome
URA3-GAP-F	GCCCAGTATTCTTAACCCAACTGCACAGAACAAAAACCTGCAGGAAACGAAGATAAATCGGGAACAAAAGCTGGAGCTCAGTTT	Amplification of P_{GAP}-*IaAO4*-T_{CYC1} expression cassette for insertion into URA3 site of yeast genome
URA3-CYC-R	TTGAAGCTCTAATTTGTGAGTTTAGTATACATGCATTTACTTATAATACAGTTTTGGCCGCAAATTAAAGCCTTCGAGCGTCC	
580-IaAO2-F	GCCCAGATCAACTAGATGGCCAAATACTCTTCGCAAGTATT	Amplification of *IaAO2* for ligation into pAN580
580-IaAO2-R	TGCTCACCATGGATCAGCTTTGTGAGGATAGAGGCG	
580-IaAO4-F	GCCCAGATCAACTAGATGGAGGTCCAAGTTGTATTGAAGGT	Amplification of *IaAO4* for ligation into pAN580
580-IaAO4-R	TGCTCACCATGGATCCAATTTCTTCACATAGAGATTGACCCCAT	
580-IaAO5-F	GCCCAGATCAACTAGATGGCCGACTTTCAAGGCTATATCA	Amplification of *IaAO5* for ligation into pAN580
580-IaAO5-R	TGCTCACCATGGATCTTTCAAAAGAAATGAATTGAGCCTAGCCACC	
580-IaCPR-F	GCCCAGATCAACTAGATGCAATCCAGCAACATCAAAGTATCT	Amplification of *IaCPR* for ligation into pAN580
580-IaCPR-R	TGCTCACCATGGATCCCACACGTCTCGCAGGT	
QIaAO2-F	GATCCTAACCACGTCGCCAA	Amplification of *IaAO2* for ligation into qPCR
QIaAO2-R	TCCAGGCAAGTCTATCGGGA	

QIaAO4-F	CCACGACTGTGCCAATGTTC	Amplification of *IaAO4* for ligation into qPCR
QIaAO4-R	CAAGCAAAGAACCACGCTCC	
QIaAO5-F	CCGGAGATAGCCAAAGAGGTC	Amplification of *IaAO5* for ligation into qPCR
QIaAO5-R	CGGATGGAGGAGGTCTAGTGTT	
QIaCPR-F	CAGATGCCGGAGAGACCTTG	Amplification of *IaCPR* for ligation into qPCR
QIaCPR-R	CCACGAACTTAGACGGGTCC	
18s-qF	GACACCCGACAAACCACAAC	Amplification of 18s for ligation into qPCR
18s-qR	CTCTAAGGGCCAATCACCAAC	

Note: "~~" stands for homology extent of each end at insert site. The italicized parts indicate 6 ×HIS tags.

TABLE S2 Plasmids and strains used in this study

Plasmid or strain	Description or relevant genotype	Source or reference
**Plasmids**		
pEASY-T5	Cloning vector with a T7 promoter, Amp^r, Kan^r	TransGen Biotech
pESC-TRP	Galactose-regulated yeast expression vector with a TRP1 selectable marker, Amp^r	Lab stock
pESC-URA	Galactose-regulated yeast expression vector with a URA3 selectable marker, Amp^r	Lab stock
pEXPR-IaAS2	Coding region of *IaAS2* cloned into pYES-DEST52, Amp^r	Lab stock
p426GPD	Yeast expression vector with a GAP promoter, Amp^r	Lab stock
Cas9-NAT	The vector with a natMX6 yeast selectable marker for expressing Cas9 protein, Amp^r	Addgene
pRS42H-gRNA-*ade*2	The vector carrying *ade*2 guide RNA of *S. cerevisiae*	Constructed by Yun (unpublished data)
pRS42H-gRNA-*leu*2	The vector carrying *leu*2 guide RNA of *S. cerevisiae*	Constructed by Yun (unpublished data)

pRS42H-gRNA-*ura*3	The vector carrying *ura*3 guide RNA of *S. cerevisiae*	Constructed by Yun (unpublished data)
*p*T5-*IaCPR*	Coding region of *IaCPR* cloned into *pEASY*-T5, Amp^r, Kan^r	This study
*p*T5-*IaAO2*	Coding region of *IaAO2* cloned into *pEASY*-T5, Amp^r, Kan^r	This study
*p*T5-*IaAO4*	Coding region of *IaAO4* cloned into *pEASY*-T5, Amp^r, Kan^r	This study
*p*T5-*IaAO5*	Coding region of *IaAO5* cloned into *pEASY*-T5, Amp^r, Kan^r	This study
pT-*IaAO2*	Coding region of *IaAO2* cloned into the *Bam*H I-*Xho* I sites of pESC-TRP, Amp^r	This study
pT-*IaAO2*-*IaCPR2*	Coding region of *IaCPR2* cloned into the *Eco*R I-*Spe* I sites of pT-*IaAO2*, Amp^r	This study
pU-*IaAO4*	Coding region of *IaAO4* cloned into the *Eco*R I-*Sac* I sites of pESC-URA, Amp^r	This study
pT-*IaAO5*	Coding region of *IaAO5* cloned into the *Eco*R I-*Sac* I sites of pESC-TRP, Amp^r	This study
pET32a-*IaCPR*	Coding region of *IaCPR* cloned into the *Eco*R I-*Sac* I sites of pET32a, Amp^r	This study
pYES-DEST52 *IaAS2*	Coding region of *IaAS2* cloned into pYES-DEST52, Amp^r	Lab stock
pT-*IaAO1*	Coding region of *IaAO1* cloned into the *Eco*R I-*Sac* I sites of pESC-TRP, Amp^r	Lab stock
GPD-*IaAO1*	Coding region of *IaAO1* cloned into the *Bam*H I-*Xho* I sites of p426GPD, Amp^r	This study
GPD-*IaAO4*	Coding region of *IaAO4* cloned into the *Bam*H I-*Xho* I sites of p426GPD, Amp^r	This study
**Strains**		
***E. coli* strains**		
*Trans*1-T1	F^-φ80(*lac*Z)△M15△*lac*X74*hsd*R(r_k^-,m_k^+)△*rec*A1398*end*A1*ton*A	TransGen Biotech
Transetta(DE3)	F^-*omp*T*hsd*S_B($r_B^-m_B^-$)*galdcmlac*Y1(DE3)pRARE(argU,argW,ileX,glyT,leuW,proL)(Cam^r)	TransGen Biotech
Transetta-pET32a-*IaCPR*	Transetta carrying pET32a-*IaCPR* plasmid	This study
***S. cerevisiae* strains**		
WAT11tfAX	WAT11*,*trp*1::P_{GAP1}-*SctHMGR1*-T_{CYC1}, *ura*3::P_{GAP1}-*ScERG20*-T_{CYC1}, *leu*2::P_{GAP1}-*SeACS*^{L641P}-T_{CYC1},*his*3::*PTEF1*-*IaAS1*-T_{CYC1}	Constructed by Yun (unpublished data)
WAT11tfA	WAT11*,*trp*1::P_{GAP1}-*SctHMGR1*-T_{CYC1}, *leu*2::P_{GAP1}-Syn*SeACS*-T_{CYC1}	Constructed by Yun

		(unpublished data)
WAT11tfAX-pT*IaAO1*	WAT11tfAX carrying pT*IaAO1* plasmid	This study
WAT11tfAX-pT*IaAO1*-pU	WAT11tfAX carrying both pT*IaAO1* and pESC-URA plasmids	This study
WAT11tfA-pD*IaAS2*	WAT11tfA carrying pD*IaAS2* plasmid	This study
WAT11tfA-pD*IaAS2*-pT	WAT11tfA carrying both pD*IaAS2* and pESC-TRP plasmids	This study
WAT11tfAX-pT*IaAO2*	WAT11tfAX carrying pT*IaAO2* plasmid	This study
WAT11tfAX-pT*IaAO2-IaCPR*	WAT11tfAX carrying pT*IaAO2-IaCPR* plasmid	This study
WAT11tfAX-pU*IaAO1*-pT*IaAO4*	WAT11tfAX carrying both pU*IaAO4* and pT*IaAO1* plasmids	This study
WAT11tfA-pD*IaAS2*-pT*IaAO5*	WAT11tfA carrying both pD*IaAS2* and pT*IaAO5* plasmids	This study
WAT11L	WAT11tfAX,*ura*3::P_{GAP1}-*IaAO4*-T_{CYC1}, *leu*2::P_{GAP1}-*IaAO1*-T_{CYC1}	This study

Urban P, Mignotte C, Kazmaier M, Delorme F, Pompon D. 1997. Cloning, yeast expression, and characterization of the coupling of two distantly related Arabidopsis thaliana NADPH-cytochrome P450 reductases with P450 CYP73A5. Journal of Biological Chemistry. 272(31);19176-19186. doi: 10.1074/jbc.272.31.19176
